# Early Detection of Acute Kidney Injury After Congenital Heart Surgery—Using Urine Proteomics to Identify New Biomarker Candidates: A Prospective Clinical Study

**DOI:** 10.3390/jcm14228253

**Published:** 2025-11-20

**Authors:** Raphael Seiler, Alexa Leona Herre, Marieluise Kirchner, Matthias Ziehm, Philipp Mertins, Felix Berger, Joachim Photiadis, Giang Tong, Liliya Brankova, Katharina R. L. Schmitt, Jana Lücht

**Affiliations:** 1 Deutsches Herzzentrum der Charité, Department of Congenital Heart Disease—Pediatric Cardiology, Augustenburger Platz 1, 13353 Berlin, Germany; 2 Deutsches Zentrum für Herz-Kreislauf-Forschung German Centre for Cardiovascular Research, 10785 Berlin, Germany; 3 Charité—Universitätsmedizin Berlin, Faculty of Medicine, Corporate Member of Freie Universität Berlin and Humboldt-Universität zu Berlin, Charitéplatz 1, 10117 Berlin, Germany; 4 Max-Delbrück-Center for Molecular Medicine in the Helmholtz Association (MDC), Proteomics Platform, 13125 Berlin, Germany; 5 Berlin Institute of Health (BIH) at Charité—Universitätsmedizin Berlin, 10178 Berlin, Germany; 6 Deutsches Zentrum für Kinder- und Jugendgesundheit (German Center for Child and Adolescent Health), Augustenburger Platz 1, 13353, Berlin, Germany; 7 Deutsches Herzzentrum der Charité, Department of Congenital and Pediatric Heart Surgery, Augustenburger Platz 1, 13353 Berlin, Germany

**Keywords:** acute kidney injury, congenital heart surgery, proteomics, urinary biomarker, critical care

## Abstract

**Background:** Acute kidney injury (AKI) is a frequent complication following congenital heart surgery and is associated with increased morbidity and mortality. Early recognition is crucial, yet standard clinical biomarkers result in delayed detection. Urine, which can be collected non-invasively, offers unique insights into kidney function and systemic responses. **Methods:** This prospective clinical study aimed to identify novel urinary biomarkers for the early detection of AKI, using high-accuracy proteome profiling. Patients with congenital heart disease undergoing cardiac surgery at Deutsches Herzzentrum der Charité were included in the study. Urine samples were collected at four timepoints: preoperatively and immediately postoperatively, and then again at six and 24 h post-surgery. Samples were analyzed using high-accuracy mass spectrometry. Linear models were applied to identify proteins associated with AKI. **Results:** A total of 67 patients with a median age of two years were included, of whom thirteen (19%) developed an AKI. Fifteen potential urinary biomarkers were identified. The most promising early indicators of AKI directly after surgery across all age groups were Chitotriosidase-1 (AUC 0.79; 95% CI:0.64–0.94), Kallikrein-1 (AUC 0.76; 95% CI:0.76–0.89), and Carbonic anhydrase 3 (AUC 0.73; 95% CI:0.6–0.87). **Conclusions**: High-accuracy mass spectrometry urine proteome profiling enabled the identification of potential new AKI biomarkers directly after congenital heart surgery. Utilization of the urinary markers Chitotriosidase-1, Kallikrein-1 and Carbonic anhydrase 3 has the potential to enable earlier detection of patients at risk for AKI. Further validation in larger, age-stratified pediatric cohorts is required to confirm the diagnostic utility of the identified urinary biomarker candidates.

## 1. Introduction

Acute kidney injury (AKI) is a frequent complication following congenital heart surgery, with an incidence rate of up to 40% [1]. It is characterized by a sudden deterioration in renal function leading to an imbalance in fluids and electrolytes, and the accumulation of nitrogenous waste products. During open-heart surgery, multiple mechanisms contribute to the pathogenesis of AKI, including ischemia–reperfusion injury, oxidative stress, acute inflammation, non-pulsatile blood flow, altered coagulation, and endothelial dysfunction. In critically ill patients, AKI is an independent predictor with increased morbidity and mortality [2]. Early diagnosis could improve therapeutic strategies and clinical outcomes; however, current diagnostic biomarkers such as serum creatinine (sCr) and cystatin C often detect AKI with significant delay [3].

Several novel AKI biomarkers, such as neutrophil gelatinase-associated lipocalin (NGAL), interleukin-18 (IL-18), liver fatty acid-binding protein (L-FABP), and kidney injury molecule-1 (KIM-1) have been proposed; however, all of these possess important limitations [4,5]. According to the Kidney Disease: Improving Global Outcomes (KDIGO) definition, AKI is diagnosed based on changes in sCr or urine output. However, sCr is an imperfect surrogate for glomerular filtration rate (GFR), particularly in critically ill patients with dynamic renal function. Its rise is delayed and influenced by age and muscle mass. In neonates, it also reflects maternal GFR [6,7]. Cystatin C may increase earlier than sCr, but its levels are affected by inflammation, steroid administration, and age [3]. Tubular injury markers such as NGAL allow for the rapid detection of renal damage but are also influenced by various pathophysiological conditions and age [8]. Consequently, the clinical applicability of all currently available biomarkers is restricted after congenital heart surgery due to their limitations.

In this study, we used an unbiased, discovery-based proteomic approach to characterize longitudinal changes in urinary protein composition. This approach was employed due to the heterogeneity in age and surgical complexity, and aimed to identify indicators of local inflammation, tubular injury and endothelial dysfunction associated with acute kidney injury (AKI). Urine was selected as the biological matrix because it is abundant and easily obtainable. It is also routinely collected during cardiac surgery and provides specific insight into both renal function and systemic physiology. Recent advances in high-resolution mass spectrometry have enabled detailed urinary proteome profiling and led to the identification of novel protein markers for kidney injury, neurological damage, and inflammatory diseases [9].

This study aimed to identify candidate biomarkers for the early detection of AKI directly after open-heart surgery in patients aged 0–65 years with congenital heart defects, using high-accuracy, mass spectrometry-based proteome profiling of urine samples collected before and after surgery.

## 2. Material and Methods

### 2.1. Study Population and Design

A prospective, single-center, observational clinical study was conducted at the Deutsches Herzzentrum der Charité between May 2020 and July 2021, following approval from the Ethics Committee of the Charité—Universitätsmedizin Berlin, Germany (approval number EA2/180/19). Furthermore, this study was preregistered with the German Register for Clinical Studies (registration number: DRKS00020885; https://drks.de/search/de/trial/DRKS00020885 (accessed on 20 August 2025). Patients were included as previously described. In short patients undergoing open heart surgery were consecutively included after informed consent was obtained. Patients with a gestational age less than 37 weeks, with syndromal diseases (e.g., Trisomy 18 or 21), known renal or urological diseases or who had experienced maternal alcohol or drug abuse were excluded [10]. To ensure data integrity, only patients with a complete set of urine samples were included in the analysis. Patients were excluded if samples were missed due to postoperative complications or secondary operations that interfered with the scheduled collection timepoints.

**Figure 1:**
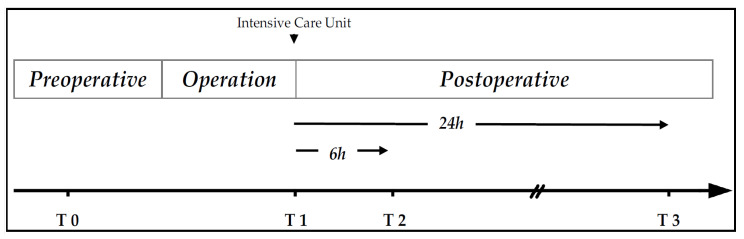
Timepoints for urine sample collection modified after Lücht et al. [10] T0 = Before surgery; T1 = immediately after surgery; T2 = 6 h after and T3 = 24 h after surgery.

Urine samples were collected at four timepoints as previously described: Before surgery (T0), immediately after surgery (T1), six hours post-surgery (T2) and 24 h post-surgery (T3) as depicted in Figure 1.

### 2.2. Outcome Measures

The primary endpoint of the study was the diagnosis of AKI according to the KIDGO classification. Secondary endpoints included the length of stay on the ICU, use of vasopressor (vasoactive-inotropic score (VIS)) and indications of organ dysfunction. In addition, explanatory variables such as fluid overload percent, duration of operation, perfusion time, re-perfusion time, cross-clamp time and vital signs were assessed. The Renal angina index (RAI) was calculated as described by Chawla et al. [11]

### 2.3. Urine Proteome Profiling

One mL urine sample was centrifuged at 1000× *g* for 5 min at 4 °C and the supernatant was collected. Protein fraction was extracted by acetone precipitation, where 4 volume ice cold acetone was added, followed by rigorous mixing of the samples and incubation at –20 °C overnight. After centrifugation at 20,000× *g* for 10 min, the supernatant was discarded, and the air-dried pellet was resolved in 8 M urea buffer (in 50 mM HEPES). Protein concentration was measured with BCA and 50 µg protein was used for further processing. Samples were reduced for 30 min in 10 mM DTT (Sigma, Hamburg, Germany) and alkylated for 45 min in 40 mM CAA (Sigma, Hamburg, Germany). 1 µg LysC (Wako Chemicals Europe, Neuss, Germany) was added for two hours, samples were diluted with three volumes 50 mM ammonium bicarbonate and digested overnight at room temperature with 1 µg sequence grade trypsin (Promega, Madison, WI, USA). Peptide samples were acidified with trifluoroacetic acid (final concentration of 1%) and cleaned using StageTip protocol [12]. Peptide samples were eluted from StageTips (80% acetonitrile, 0.1% formic acid), and after evaporating the organic solvent, peptides were resolved in sample buffer (3% acetonitrile/0.1% formic acid). For each sample 1 µg of peptide was separated on a 20 cm reversed-phase column (75 µm inner diameter, packed with ReproSil-Pur C18-AQ (1.9 µm, Dr. Maisch GmbH, Ammerbuch, Germany) using a 98 min gradient with a 250 nl/min flow rate of increasing Buffer B concentration (from 2 to 60%) on a High-Performance Liquid Chromatography (HPLC) system (Thermo Fisher Scientific, Waltham, Massachusetts, USA). Peptides were measured on a Q Exactive HF-X Orbitrap instrument (Thermo Fisher Scientific, Waltham, Massachusetts, USA). The mass spectrometer was operated in the data dependent mode with a 60 K resolution, 3 × 10^6^ ion count target and maximum injection time 10 ms for the full scan, followed by Top 20 MS2 scans with 15 K resolution, 1 × 10^5^ ion count target and maximum injection time of 22 ms. Dynamic exclusion was set to 30 s.

Raw data were processed using MaxQuant software package (v1.6.10.34) and a decoy human UniProt database (HUMAN.2019-07), containing forward and reverse sequences. The search included variable modifications of oxidation (M), N-terminal acetylation, deamidation (N and Q), and fixed modification of carbamidomethyl cysteine. Minimal peptide length was set to six amino acids, and a maximum of two missed cleavages was allowed. The FDR was set to 1% for peptide and protein identifications. Unique and razor peptides were considered for quantification. MS2 identifications were transferred between runs with the “Match between runs” option. The integrated LFQ (label-free) quantitation algorithm was applied. No additional data normalization was applied. Reverse database hits, potential contaminants (except Albumin), and proteins only identified by site, that is, proteins identified only by modified peptides were excluded. LFQ values were log2-transformed, and missing values were imputed by random draw from the Gaussian distribution with 0.3* standard deviation and downshift of 1.8* standard deviation of the observed values per sample

### 2.4. KDIGO Criteria

AKI stage I: Serum creatinine concentration: 1.5 to 1.9-fold within 7 days or ≥0.3 mg/dL within 48 h; Urine excretion: <0.5 mL/kg/h over a period >6 h.

AKI stage II: Serum creatinine concentration: 2 to 2.9-fold; Urine excretion: <0.5 mL/kg/h over a period >12 h.

AKI stage III: Serum creatinine concentration: ≥3-fold or sCr. ≥4 mg/dL with acute increase ≥0.5 mg/dL; Urine excretion: <0.3 mL/kg/h over a period >24 h or anuria >12 h.

AKI stage was adjudicated by a pediatric cardiologist blinded to proteomics results.

### 2.5. Statistical Analysis

Descriptive and comparative statistics were conducted using IBM SPSS Statistics (version 29.0.2, IBM Corp., Armonk, NY, USA). Numbers are presented as counts and percentages for categorical data and median with interquartile range (IQR) [Q1–Q3] for continuous data. The two-sided Mann–Whitney test and the two-sided Chi-squared test was used where appropriate to compare clinical data of AKI versus non-AKI patients. Statistical analyses of proteomics data were performed using R (version 4.0.3, R Foundation for Statistical Computing, Vienna, Austria). Comparisons between different timepoints were performed separately for cases with AKI and without AKI, using two-sided paired *t*-test. Proteins passing the FDR-based significance cut-off of 5% were considered differentially abundant. Enrichment analysis using Fisher’s exact test for increased or decreased proteins using GO cellular component (CC) annotations without IEA evidence was performed with minimum 3 proteins per term and 5% FDR cut-off.

To identify markers associated with AKI, sCr difference and sCr ratio were analyzed as continuous variable using linear models accounting for sex and age as co-factors using the limma R package (version 3.50.3) (trend = T, robust = T). Because of the limited number of AKI cases (n = 13), we refrained from multivariable modeling beyond age and sex to avoid overfitting. According to KDIGO AKI definition, sCr difference and sCr ratio were calculated as follows. SCr difference = max (sCr@T1, sCr@T2, sCr@T3)-sCr@T0); sCr ratio = max (sCr@T1, sCr@T2, sCr@T3)/sCr@T0. Proteomics data for different timepoints were analyzed separately excluding proteins with <50% valid values at that timepoint. Proteins with two-sided ≤5% FDR are deemed hits. Hits from all timepoints and both continuous variables were taken together as candidate set (98 proteins). Among these, proteins were further highlighted when showing in the categorical comparison at T1 between AKI cases and non-AKI cases an absolute log2 fold-change ≥ B2M (a previously established marker) and a nominal *p*-value < 0.05 in a two-sided Welch *t*-test. *p*-values are multiple testing corrected by the Benjamini–Hochberg method except if denoted differently.

Receiver operating characteristics were calculated using the ROCR (version 1.0-11) and pROC R (version 1.18.0) package based on predictions from a logistic regression model. The model was fitted in R using a binomial family generalized linear model (GLM), with AKI as the outcome and log2 protein quantification as predictor.

## 3. Results

### 3.1. Patient Characteristics

A total of 67 patients (25 females, 37.3%) undergoing open heart surgery were included over a twelve-month period. Of these patients, 13 (19%) developed an AKI of any stage according to KDIGO criteria. The median age at the time of the operation was two years [IQR 0–5]. No significant differences were observed in terms of age at the time of the operation, sex, height and weight between the AKI and the non-AKI patients. However, there was an overrepresentation of neonates in the AKI group (*p* < 0.05). Clinical characteristics and perioperative data are summarized in Table 1.

### 3.2. Perioperative Data and Outcome

In comparison to the non-AKI group, patients with AKI underwent longer operations (*p* = 0.001), had longer cardiopulmonary bypass (CPB) durations (*p* = 0.001), and had longer stays in the ICU and hospital (*p* = 0.001). In addition, AKI patients also had a longer duration of mechanical ventilation (*p* = 0.001), required more packed red blood cell transfusions, and exhibited a higher need for inotropic support (*p* = 0.001), as indicated by a higher VIS.

Baseline eGFR did not differ between AKI and non-AKI patients. However, postoperative eGFR was significantly lower at all timepoints in AKI patients (T1 *p* = 0.04; T2 *p* = 0.003; T3 *p* = 0.001). We observed no clinically relevant differences in sodium or potassium levels between groups. A trend towards higher blood glucose levels was noted in patients with AKI; however, this did not reach statistical significance (Table 2).

There were no in-hospital death in the entire cohort (Table 1).

### 3.3. Urine Proteomics in the Context of Heart Surgery

#### Coverage

**Figure 2:**
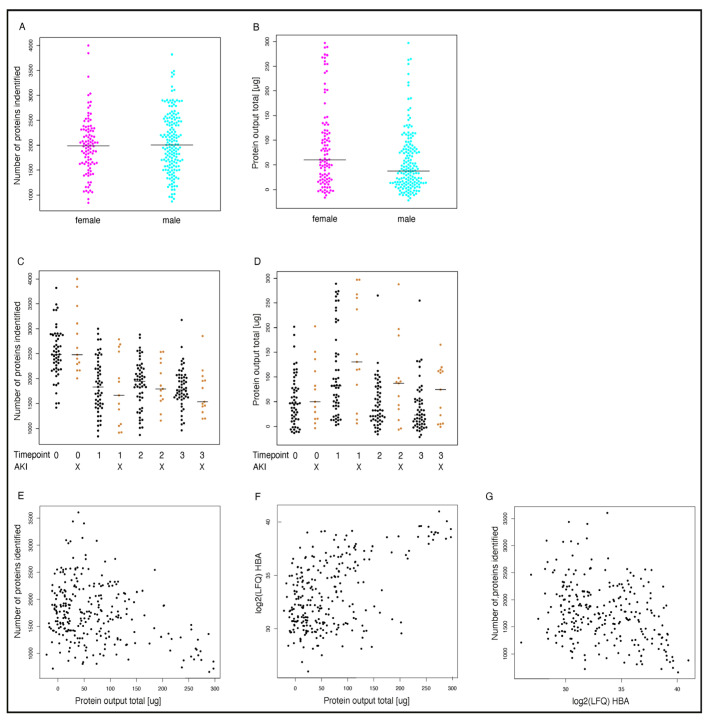
Protein coverage. Number of identified proteins and total protein output are displayed in relation to sex (**A**,**B**) perioperative timepoint/AKI status (AKI patients n = 13; non-AKI patients n = 54, bars represent median per group) (**C**,**D**) and Hemoglobin A abundance (**F**,**G**). Panel (**E**) depicts the relation of the number of identified proteins to total protein amount. Timepoints (0: Preoperative; 1 = direct after surgery; 2 = 6 h after surgery, 3 = 24 h after surgery); HBA = Hemoglobin A. AKI = Acute kidney injury.

We identified approximately 2500 different proteins in the urine of patients preoperatively and around 1900 postoperatively. We found that the rate of protein identification was lower in postoperative samples at all timepoints (Figure 2C), accompanied by an increase in total protein concentration after surgery (Figure 2D). These elevated postoperative protein concentrations correlate with a notably high abundance of typical blood proteins, such as Hemoglobin A (Figure 2F,G). It is well established that blood proteins significantly impact the dynamic range of urine sample proteome [13], resulting in decreased proteome coverage. In our study, a higher urine protein concentration was associated with a lower ID rate and a higher detection rate of typical blood proteins (Figure 2E–G). This effect was independent of sex and AKI status (Figure 2A,B).

### 3.4. Clustering and Proteome Changes upon Surgery

**Figure 3:**
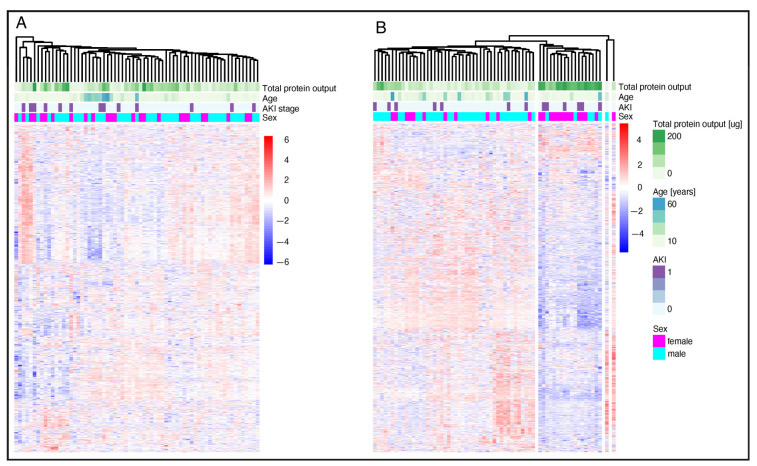
Cluster and proteome change upon surgery. Heatmap of relative protein abundance preoperative (**A**) and direct after surgery (**B**). AKI = Acute kidney injury. AKI patients n = 13; non-AKI patients n = 54. Proteins are z-scored the heatmap. Proteins and samples are clustered by hierarchical UPGMA clustering.

**Figure 4:**
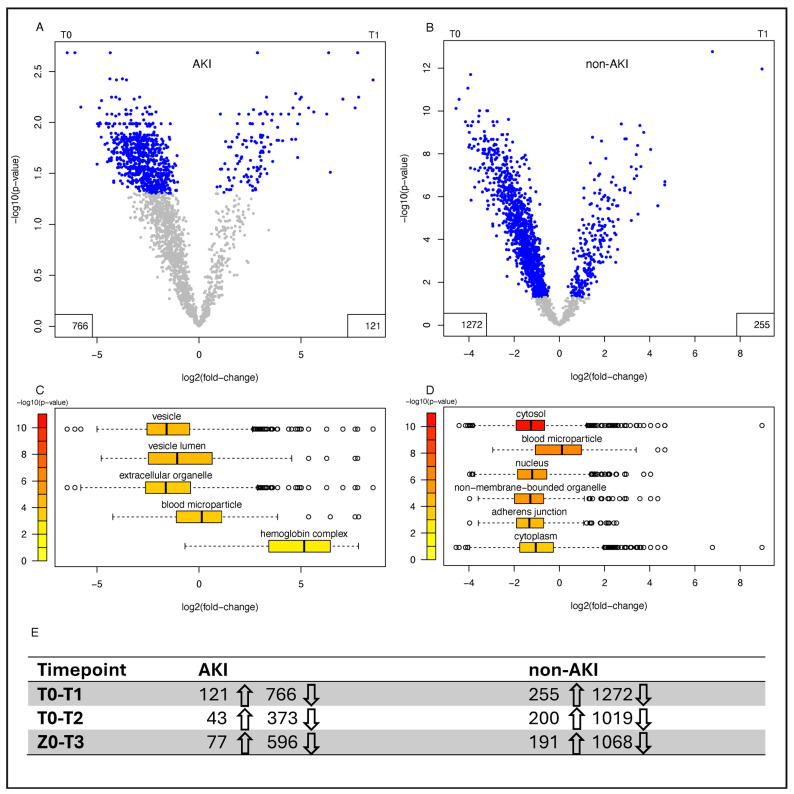
Changes in the proteome following surgery in patients with and without AKI. Relative changes in the urine proteome from T0 to T1 are displayed for AKI (n = 13) (**A**) and non-AKI (n = 54) (**B**) patients. Coloring cut-off = *p* < 0.05. The number of significant upregulated (↑) and downregulated proteins (↓) for each timepoint compared to timepoint 0 are stated in panel **(E).** Boxplot distributions of protein abundances from significantly enriched cellular components from a Gene Ontology (GO) term enrichment analysis for AKI (n = 13) (**C**) and non-AKI patients (n = 54) (**D**). Boxplot limits defined as center line: median, box limits: upper and lower quartiles, whiskers: most extreme data point which is no more than range times the interquartile range from the box, points: outliers. AKI = Acute kidney injury.

Preoperative urine samples did not cluster within patient subgroups based on clinical parameters (Figure 3A). However, immediately after surgery (T1) patients with a higher total protein output did cluster, and there was an increased occurrence of AKI. (Figure 3B). Next, we investigated changes in urine composition post-surgery at different timepoints compared to the pre-surgery state (Figure 4). Significant changes were observed in both AKI and non-AKI cases, with the greatest differences occurring at timepoint T1 (Figure 4A,B). Enrichment analysis of significantly different proteins showed that “blood microparticle” and “hemoglobin complex” proteins were enriched after surgery (T1), particularly strong in AKI cases (Figure 4C,D). Interestingly, these changes appear to be long-lasting, as several hundred proteins still show differential abundance (T1 > T3 > T2) in both patient subgroups at timepoint T3 (see Supplementary Figure S1).

### 3.5. Selection of AKI Biomarker Candidates from Urine Proteome Profiles

**Figure 5:**
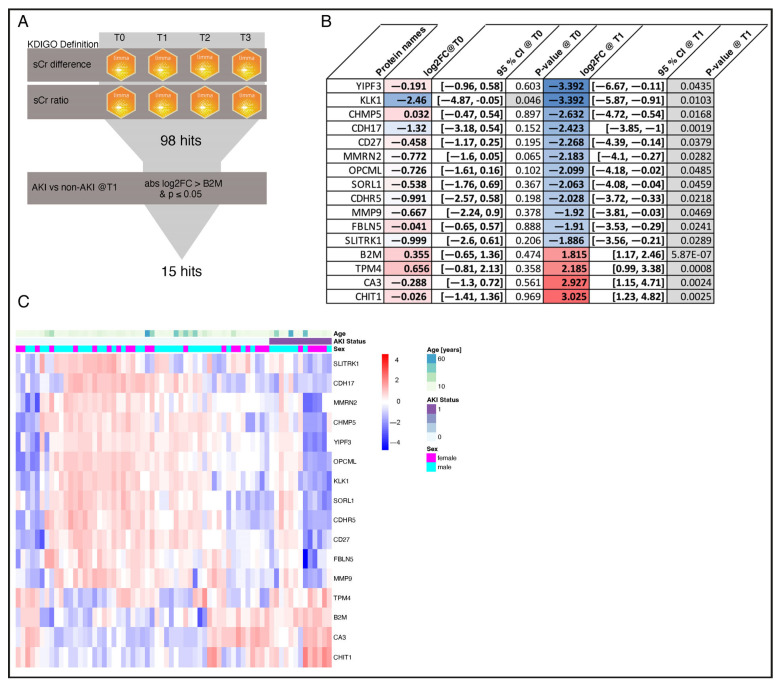
Protein selection. The applied protein selection process is illustrated in flowchart (**A**). The accordingly selected AKI candidate markers, including beta-2 microglobulin as a reference, are displayed with their relative abundance direct after surgery (T1) in heatmap (**C**) and ranked based on their log2 fold changes for AKI (n = 13) vs. non-AKI (n = 54) patients direct after surgery (T1) in (**B**). Red colored = elevated in AKI patients; blue colored = reduced in AKI patients. Proteins are z-scored in the heatmap. Proteins and samples within AKI status groups are clustered by hierarchical UPGMA clustering. AKI = Acute kidney injury; KDIGO = Kidney Disease: Improving Global Outcomes; sCr = Serum creatinine; log2FC = Log base 2 fold change; YIPF3 = Protein YIPF3; KLK1 = Kallikrein-1; CHMP5 = Charged multivesicular body protein 5; CDH17 = Cadherin-17; CD27 = CD27 antigen; MMRN2 = Multimerin-2; OPCML = Opioid-binding protein; SORL1 = Sortilin-related receptor; CDHR5 = Cadherin-related family member 5; MMP9 = Matrix metalloproteinase-9; FBLN5 = Fibulin-5; SLITRK1 = SLIT and NTRK-like protein 1; B2M = Beta-2 microglobulin; TPM4 = Tropomyosin alpha-4 chain; CA3 = Carbonic anhydrase 3; CHIT1 = Chitotriosidase-1. CI = Confidence interval.

**Figure 6:**
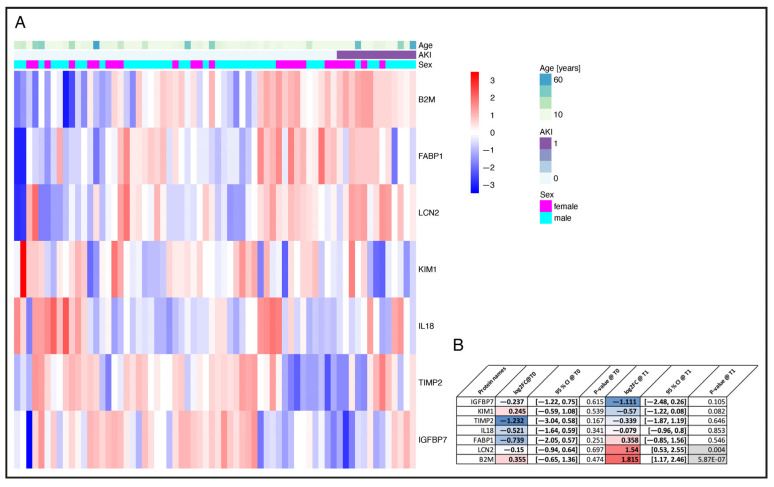
AKI reference marker. Relative abundance of the identified known AKI marker direct after surgery (T1) (**A**). Proteins are z-scored in the heatmap. Proteins and samples within AKI status groups are clustered by hierarchical UPGMA clustering. In panel (**B**) log2 fold changes preoperatively (T0) and direct after surgery (T1) are displayed with respective *p*-values for the identified AKI marker. Red colored = elevated in AKI patients; blue colored = reduced in AKI patients AKI = Acute kidney injury; B2M = Beta-2 Mikroglobulin; FABP1 = Liver type Fatty acid-binding protein; LCN2 = NGAL, Neutrophil gelatinase-associated lipocalin; KIM1 = Kidney injury molecule 1; IL 1 = Interleukin 18; TIMP2 = Tissue inhibitor of metalloproteinase 2; IGFBP7 = Insulin-like growth factor-binding protein 7; log2FC = Log base 2 fold change. AKI patients n = 13; non-AKI patients n = 54. CI = Confidence interval.

Since sCr rises late and often discreetly, especially in subclinical AKI, relying on strict sCr cut-offs to analyze alteration in urinary protein abundance in AKI and non-AKI patients may be insensitive to discover AKI biomarker candidates. Therefore, we analyzed the patients’ individual sCr courses with limma as a continuous variable to identify potential urinary protein markers for AKI risk immediately after surgery (Figure 5A). A total of 98 different proteins significantly associated with the individual sCr course of each patient across all timepoints were identified (see Supplementary Table S1). Our measurements included several previously described urinary AKI biomarker candidates [4]: B2M, NGAL, KIM-1, L-FABP, IGFBP7, and TIMP2 (Figure 6). Of these biomarkers, B2M showed the best performance in terms of differential abundance between AKI and non-AKI patients. To further select the most relevant proteins for early AKI detection, we used the absolute log2 fold change of B2M at T1 in AKI versus non-AKI patients and a nominal *p*-value of ≤0.05 was used as a cut-off. Abundance profiles for the resulting 15 top regulated proteins are shown in Figure 5C. Additionally, the latter candidate markers were ranked by effect size (log2 fold change in AKI versus non-AKI patients at T1) (Figure 5B).

**Figure 7:**
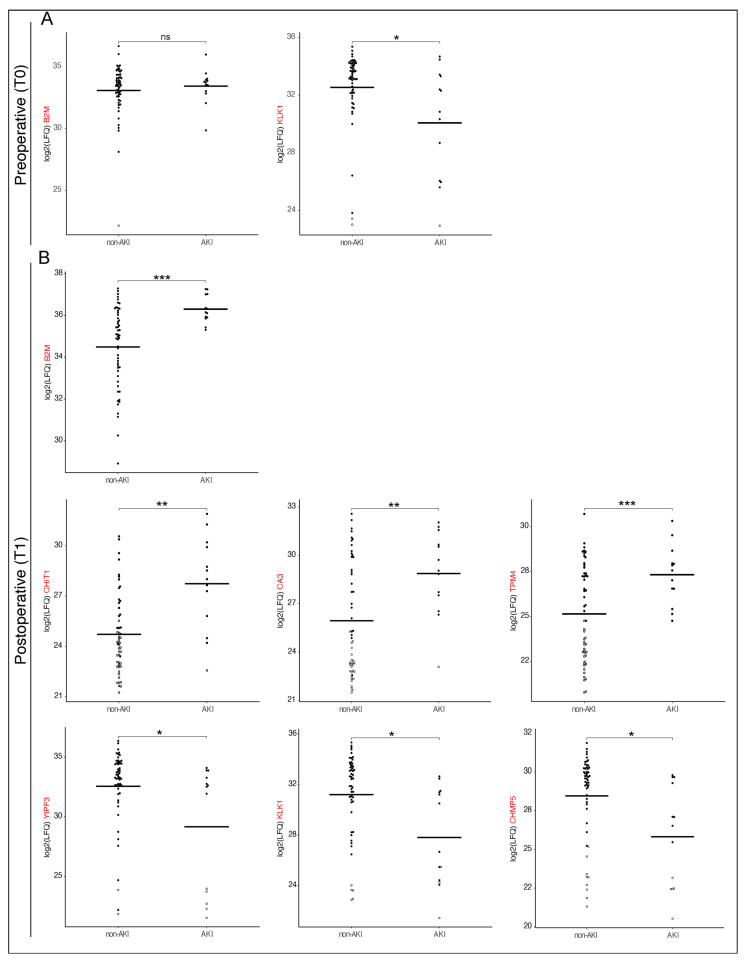
Dynamic Shifts in Biomarker abundance: Preoperative vs. Postoperative Differences in AKI and non-AKI patients. Different preoperative (**A**) and postoperative (**B**) log2 fold changes in biomarker abundance between AKI (n = 13) and non-AKI (n = 54) (**B**) patients are shown; bars representing median per group. Filled circles represent measured values; hollow circles indicate imputed values, and horizontal lines denote mean values. Statistical significance is indicated as follows * < 0.05, ** < 0.01, *** < 0.001 nominal *p*-value. Log2FC = Log base 2-fold change; B2M = Beta-2 microglobulin; KLK1 = Kallikrein-1; CHIT1 = Chitotriosidase-1; CA3 = Carbonic anhydrase 3; TPM4 = Tropomyosin alpha-4 chain; YIPF3 = Protein YIPF3; CHMP5 = Charged multivesicular body protein 5.

The selected proteins include transmembrane transporters and receptors, enzymes, and proteins involved in cellular trafficking, endothelial function, cell growth and differentiation, inflammation, the immune response, the coagulation system, and the extracellular matrix. While most of the identified proteins were found to be down-regulated in AKI patients, Chitotriosidase-1 (CHIT1), Carbonic anhydrase 3 (CA3), and Tropomyosin alpha-4 chain (TPM4) showed increased abundance at T1 (Figure 7). CHIT1 exhibited the most significant difference between AKI and non-AKI patients with a log2 fold change of 3.03 (95% CI: 1.23–4.82, nominal *p* = 0.003) at T1. Consistent with AKI pathophysiology, CHIT1 is associated with systemic inflammation and a reaction to oxidative stress, and is involved in several infectious, allergic, and autoimmune conditions [14]. CA3 catalyzes the reversible conversion of carbon dioxide and water into carbonic acid, and has the lowest hydratase activity of the CA isoforms. It was found to be upregulated in AKI patients with a log2 fold change of 2.93 (95% CI: 1.15–4.71, nominal *p* = 0.002). CA isoenzymes play a crucial role in maintaining water and salt homeostasis by regulating sodium transport and oxygen consumption in renal tissue [15]. TPM4, one of the top regulated proteins, was detected in the urine of AKI patients with a 2.19 log2 fold change (95% CI: 0.99–2.46, nominal *p* = 0.001). TPM4 is an actin-binding protein and a member of the tropomyosin family of proteins, which regulates muscle contraction and stabilizes actin filaments in both muscle and non-muscle cells. TPM4 is also involved in cellular stress responses, stabilizing actin filaments to maintain cellular structures [16,17]. By contrast, Protein YIPF3 (Protein YIPF3) with a log2 fold change of −3.39 (95% CI: −6.67–−0.11, nominal *p* = 0.04), Kallikrein-1 (KLK1) with a log2 fold change of −3.39 (−5.87–−0.91, nominal *p* = 0.01) and Charged multivesicular body protein 5 (CHMP5) with a log2 fold change of −2.63 (95% CI: −4.72–−0.54, nominal *p* = 0.017) showed the lowest average abundance in AKI patients compared to non-AKI patients. YIPF3 plays a role in the regulation of intracellular membrane trafficking, particularly within the endoplasmic reticulum and Golgi apparatus [18]. KLK1 is known to be activated during open heart surgery and is involved in activation of plasmatic coagulation. Interestingly, KLK1 is the only one of the top 15 selected proteins for which there is a significant difference between the AKI and non-AKI patients preoperatively at T0. Patients who developed AKI after surgery were found to have a log2 fold change of −2.46 (95% CI: −4.87–−0.05, nominal *p* = 0.04) preoperatively for KLK1, compared to non-AKI patients. CHMP5 plays a significant role in maintaining cellular homeostasis and the proper functioning of intracellular trafficking systems by regulating protein sorting and degradation, membrane remodeling and cell division, for example in the trafficking of aquaporin-2 in the collecting duct of the kidneys [19].

**Figure 8:**
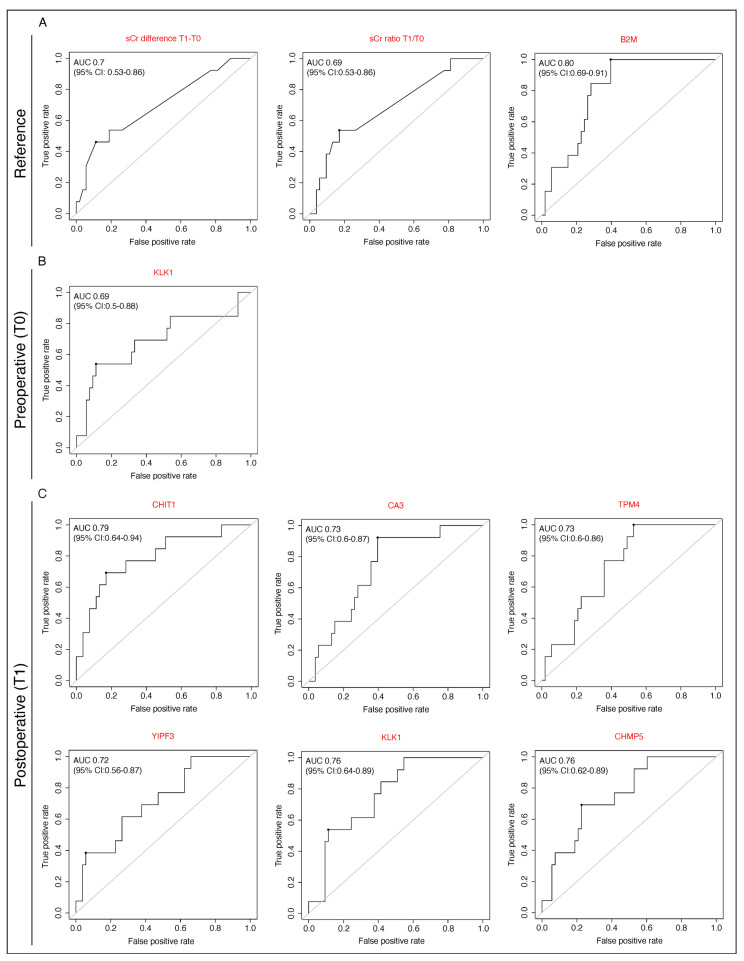
Performance of selected potential urinary AKI biomarkers. Receiver operating curves (ROC) including areas under the curve values (AUC) are displayed for AKI reference biomarker (**A**) and new AKI biomarker candidate preoperative (T0) (**B**) and postoperative (T1) (**C**). AKI = Acute kidney injury; KDIGO = Kidney Disease: Improving Global Outcomes; sCr = Serum creatinine; B2M = Beta-2 microglobulin; KLK1 = Kallikrein-1; CHIT1 = Chitotriosidase-1; CA3 = Carbonic anhydrase 3; TPM4 = Tropomyosin alpha-4 chain; YIPF3 = Protein; CHMP5 = Charged multivesicular body protein 5.

Figure 8C shows the receiver operating characteristic (ROC) curves for the top 6 candidate markers at T1 (immediately after surgery). These demonstrate good performance across all age groups, with an area under the curve (AUC) value of 0.79 (95% CI: 0.64–0.94, CHIT1), 0.73 (95% CI: 0.6–0.87, CA3) and 0.73 (95% CI: 0.6–0.86, TPM4) for three individual proteins with high abundance in AKI patients and an AUC of 0.72 (95% CI: 0.56–8.87, YIPF3), 0.76 (95% CI: 0.64–0.89, KLK1) and 0.76 (95% CI: 0.62–0.89, CHMP5), respectively, for proteins with low abundance in AKI patients. The AUC of KLK1 for AKI detection preoperatively was 0.69 (95% CI: 0.5–0.88) (Figure 8B). In addition, Figure 8A shows the AUC for B2M at T1, the sCr ratio T0/T1 and the sCr difference T0–T1, according to the KDIGO AKI definition routinely used Biomarker in everyday clinical practice. Thus, the presented data clearly shows the identified potential of new AKI biomarker exceeding AUC performance of sCr and is at least equal to B2M.

## 4. Discussion

Acute kidney injury after congenital heart surgery is a common complication and associated with elevated morbidity and mortality [20]. With an incidence of 20%, AKI was frequent in our heterogeneous cohort, however this was slightly lower than the previously reported AKI incidence of 30–40% after congenital heart surgery [21]. Nevertheless, patients with AKI experienced significantly more postoperative complications than those without AKI. Analysis of clinical data, including length of stay in intensive care, administration of erythrocyte concentrate, duration of ventilation, and the requirement for catecholamines to stabilize circulation, indicated that patients with AKI in our cohort were considerably more ill. These results are consistent with previous studies reporting poorer outcomes for patients with AKI following cardiac surgery [1,20,21,22]. Improving outcomes following congenital heart surgery requires the early identification of patients at risk of AKI. Unfortunately, this is often hindered by the delayed response of the functional biomarkers that are routinely used in clinical practice, such as sCr and Cystatin C. Recently, there has been extensive discussion about new biomarkers that can indicate kidney injury before a decline in the glomerular filtration rate occurs [23]. However, significant diagnostic challenges remain, largely due to the heterogeneity of patients with congenital heart defects and the absence of age-specific cut-off values, as well as the specific limitations of each biomarker. Due to the limited blood supply, particularly in neonates and small infants, we aimed to analyze patients’ urine to identify new AKI biomarkers candidates following cardiac surgery. This approach is intended to mitigate iatrogenic anemia and minimize the need for painful procedures associated with blood collection. Moreover, urine is easy to collect, non-invasive, and provides unique insights into kidney function and patients’ systemic status. Approximately 70% of the urine proteome is secreted by the renal tubular system, while the remaining 30% is filtered from blood plasma [24]. Using high-accuracy mass spectrometry to profile the urine proteome, enables the discovery of multiple potential AKI biomarkers. This approach helps overcome the limitations associated with conventional single-hypothesis-testing approaches. Using this method, we detected approximately 2500 unique urinary proteins preoperatively and around 1900 unique urinary proteins postoperatively. Accordingly, postoperative coverage was markedly lower (approximately 24%). However, total protein output was elevated in postoperative urine samples and was clearly correlated with the very high abundance of typical blood proteins. Blood proteins are known to have a significant impact on the dynamic range of the urine sample proteome, resulting in the observed decrease in postoperative protein coverage [25]. Furthermore, haemolysis is common in patients after CPB, particularly in neonates and young infants [26], and the levels of haemolysis are closely associated with the incidence and severity of AKI after cardiac surgery [27]. This explains why at T1, a cluster of AKI patients was visible in the urine proteome, mainly due to the higher protein output and therefore, most likely higher amount of haemolysis in AKI patients during CPB. Consistent with this observation, AKI patients in our cohort were more likely to receive packed red blood cells than non-AKI patients during the postoperative period.

For both, AKI and non-AKI patients, the greatest difference in urine proteome composition compared to preoperative urine was detected immediately after surgery (T1) with a smaller difference at six hours postoperatively, followed by a moderate increase in differential abundance 24 h after surgery. Since fluid balance is altered directly after open heart surgery and patients often suffer from intravascular fluid deficiency, a lot of fluids have to be administered during the first 24 postoperative hours, which may result in diluted urine. Furthermore, the administration of diuretic drugs during the postoperative period may also have impacted urine composition. In addition to the pronounced differences observed at T1, it is crucial to detect AKI as early as possible, thus, we focused the further analysis mainly on T1.

Over the past decade, many new urinary AKI biomarkers (uNGAL, uIL-18, uL-FABP, and uKIM-1, for example) have been described by other to exhibit moderate to good performance in detecting AKI after cardiac surgery in selected patient cohorts [4,5]. In the present study, these proteins were reliably detected, but there was little significant difference in their regulation between AKI and non-AKI patients. This may be explained by the fact that our study included a highly heterogenous patient cohort of all ages, from newborns to adults. Pediatric renal physiology differs fundamentally from that of adults. Nephrogenesis is completed by approximately 34–36 weeks of gestation, and postnatal maturation of both glomerular filtration and tubular transport continues throughout infancy. Consequently, baseline glomerular permeability is higher and tubular reabsorption processes are age-dependent, influencing urinary protein excretion and thereby affecting biomarker concentrations, particularly in neonates and young infants. Furthermore, age-related differences in body water distribution and the use of pediatric-specific equations for estimating glomerular filtration rate further highlight the physiological differences between pediatric and adult patients [28,29,30]. Moreover, as previously discussed, our analysis focused on urine collected immediately after surgery, whereas most previously described biomarkers were shown to increase four to 24 h after surgery [4,5]. A recent meta-analysis of AKI biomarker performance after cardiac surgery found that only uNGAL showed good diagnostic accuracy four to six hours postoperatively in pediatric patients. However, due to potential comorbidities such as diabetes and pre-existing kidney dysfunction, the performance of uNGAL after cardiac surgery in adult patients was limited [4]. Similarly, urinary KIM-1, uIL-18, and uL-FABP did not discriminate between AKI and non-AKI patients in our cohort, whereas uNGAL showed modest diagnostic accuracy across all age groups. Taken together, the heterogeneity of our cohort and the earliest urine collection after surgery may explain the modest performance of the AKI biomarkers described to date in our cohort. While this finding is negative for these specific markers, we argue that it highlights a critical diagnostic gap. The inability of established markers to detect AKI in the immediate post-operative period highlights the clinical need for new biomarkers that can detect AKI earlier—this is the central motivation and primary objective of our study.

According to the KDIGO definition, AKI is characterized by an increase in sCr and/or a decrease in urine output. Unfortunately, sCr is known to elevate late in AKI [31]. Therefore, discovering new AKI biomarker candidates using strict sCr cut-off for the AKI definition may be insensitive. First we analyzed patients’ individual sCr courses from the preoperative baseline value across the different postoperative timepoints as a continuous variable in order to identify urinary proteins associated with AKI. 98 different proteins were found to be significantly associated with changes in sCr levels at different timepoints. In a second step, we aimed to assess the ability of the identified potential candidate marker to differentiate between AKI and non-AKI patients according to the KDIGO definition. Urinary B2M, a previously described marker of tubular damage, was significantly elevated in AKI patients compared to non-AKI patients with a log2 fold change of 1.81 (*p* < 0.001) and an AUC of 0.8. This is consistent with recent results from Barton et al., who reported a moderate ability of urinary B2M to detect patients with AKI in a retrospective study of pediatric patients over one year of age [32]. Thus, to further select the most relevant candidate proteins, the B2M log2 fold-change value was used as a cut-off and 15 higher regulated proteins in AKI patients were analyzed further. Among these, we identified proteins produced both systemically and locally in the kidney, indicating systemic inflammation, oxidative stress, tissue ischemia, endothelial dysfunction, and altered coagulation status. Most of the identified proteins were found to be significantly less abundant, while CHIT1, CA3, and TPM4 were found to be upregulated in AKI patients. CHIT1 is an enzyme involved in the degradation of chitin. It is mainly produced by activated macrophages and epithelial cells and its role in various inflammatory and fibrotic conditions, including kidney diseases, has been studied [14]. CHIT1 has recently been identified as a marker of macrophage activation in chronic kidney diseases and type 2 diabetes mellitus, as well as bronchial asthma. In acute coronary syndrome, an association was found between plasma CHIT1 activity and the inflammatory response [33]. In AKI, inflammation, immune cell infiltration and oxidative stress are significant contributors to kidney damage, and elevated CHIT1 levels could be linked to the inflammatory response in this context.

Carbonic anhydrase plays a vital role in both the onset and recovery phases of acute kidney injury, particularly through its regulation of sodium transport and oxygen consumption in renal tissue [34]. However, CA3, the isoenzyme with the lowest hydratase activity, is known to function in an oxidative environment and is only abundantly expressed in the cytosol of skeletal muscle cells, adipocytes, and hepatocytes [35]. Gailly et al. reported that CA3 induction was associated with increased cell proliferation and oxidative stress in their mouse model of proximal tubule dysfunction (an established model for Dent’s diseases). They also stated that CA3 upregulation may be part of the cellular response to oxidative stress. Accordingly, they found CA3 to be upregulated in mice with proximal tubule damage and in patients with Dent’s disease. Therefore, urinary CA3 may serve as an early AKI biomarker after cardiac surgery, indicating oxidative stress. To the best of our knowledge, research exploring the connection between TPM4 and AKI is lacking. However, the involvement of actin-binding proteins, such as tropomyosin, in cellular stress responses and structural integrity is well established. Tropomyosin stabilizes actin filaments, which are crucial for maintaining cellular structures. Dysregulation of these processes may influence how cells respond to injury, including that seen in kidney damage [16,17]

As previously mentioned, most of the candidate markers identified were found to be present in reduced abundance in the urine of AKI patients after cardiac surgery. The most promising of these are KLK1, YIPF3, and CHMP5. KLK1 is a serine protease that is expressed in various tissues, including renal tissue. It processes kininogen substrates to release vasoactive kinin peptides. It is known to be activated during open-heart surgery [36]. Furthermore, KLK1 exhibits pleiotropic effects in protecting against oxidative damage to the heart, kidneys, and brain [37]. Therefore, lower urinary levels may indicate more pronounced oxidative stress and/or a reduced abilita to respond to oxidative stress in AKI patients. Interestingly, in the present study, lower urinary KLK1 levels preoperatively were significantly associated with a higher risk of AKI. The AUC at T0 (before surgery) was 0.69, which is fairly good and could be even improved by combining it with clinical risk assessment tools such as the renal angina score [11]. Importantly, this approach would enable identification of patients at increased risk of AKI already in the preoperative phase. This early risk stratification could support targeted preventive measures—such as minimizing avoidable perioperative stressors and applying the KDIGO care bundle—to reduce the likelihood of postoperative AKI [38]. YIPF3 is a protein that plays a role in regulating intracellular membrane trafficking, particularly within the endoplasmic reticulum and the Golgi apparatus [18]. Autophagic processes, which clear damaged organelles and proteins, are critical during kidney stress. Disruption to the trafficking machinery (potentially involving YIPF3) may impair autophagy, and contribute to AKI progression. By maintaining proper protein trafficking, YIPF3 could influence cell stress response pathways to prevent excessive apoptosis. However, to the best of our knowledge YIPF3 has not yet been linked to AKI. Similarly, CHMP5, as part of the ESCRT (endosomal sorting complex required for transport) system, may be involved in regulating apoptosis and the cellular stress response during AKI development [39,40]. Inhibiting it can activate apoptotic and necrotic cell death pathways, which are relevant in various conditions including acute kidney injury. Through ESCART III, CHMP5 is involved in Aquaporine-2 trafficking in the collecting duct of the kidney, which is a key regulator in water and salt homeostasis [19].

The exact mechanisms by which the aforementioned proteins accumulate or decrease during AKI, and their relationship to the pathophysiology of AKI remain to be elucidated. Further studies are therefore warranted to confirm these promising results

Our findings highlight the potential clinical utility of early urinary biomarkers such as CHIT1, KLK1, and CA3. Their preoperative and early postoperative differential abundance may reflect renal stress before overt functional decline, providing an opportunity to identify patients at increased risk in a timely manner. Incorporating these biomarkers into perioperative monitoring could complement the existing KDIGO criteria and support the management of fluids and medications on an individual basis. Ultimately, this could facilitate earlier and more targeted renoprotective interventions in pediatric cardiac surgery.

## 5. Limitations

The findings of a single-center study may not be universally applicable due to differences in patient demographics, surgical techniques, and postoperative care protocols between different cardiac centers. Selection bias or institution-specific practices may have influenced the patient cohort in the present study. Furthermore, the cohort was relatively small and the number of AKI cases was limited. This impacts the statistical power and may prevent subtle but relevant differences from being detected. Additionally, as patients of all ages were included, age-specific differences in the urine proteome are possible confounders. To address this, preoperative urine was analyzed, and relative changes across postoperative timepoints were investigated using the preoperative timepoint as an individual baseline value. Routine renal ultrasound was not performed, which may have limited our ability to identify structural or obstructive causes of renal dysfunction. Additionally, several unmeasured perioperative variables, such as fluctuations in haemodynamic stability, exposure to nephrotoxic medications and differences in fluid management strategies, may have contributed to the observed variability in AKI incidence and biomarker expression. These parameters were not systematically captured in our dataset, so they represent potential confounders that could influence renal perfusion and the release of injury-related proteins.

Due to the limited serum volume available from our pediatric cohort, we prioritized samples for extensive parallel multiplex analysis (Luminex) of SIRS and capillary leak biomarkers [10]. Consequently, it was not feasible to make an additional comparison with serum cystatin C, which we believe would have been important for directly comparing its AKI detection capabilities against sCr, as it would have required a higher sample volume for ELISA. This comparison remains an objective for future validation studies.

High-accuracy mass spectrometry urine proteome profiling achieved good protein detection across all timepoints, although postoperative coverage was reduced due to higher blood content, which was even more pronounced in AKI patients. Therefore, due to the technical limitations of this method, some proteins of interest may have been overlooked. The aim of the present study was to discover potential AKI biomarkers candidates using a discovery-based approach to profile molecular alterations on a proteomic scale. The most promising candidates require validation in well-powered studies. Furthermore, a cell culture model utilizing kidney cells will be used to conduct interventions to regulate the promising candidate proteins. This will mark the subsequent stage prior to the initiation of a clinical intervention study.

## 6. Conclusions

This study employed a urine-based approach to enable the non-invasive detection of early renal injury, which is particularly advantageous in neonates and infants with limited blood volume. Biomarker discovery focused on a very early postoperative time window using urine collected immediately after surgery, before the clinical manifestation of acute kidney injury (AKI). Furthermore, the analysis was driven by continuous changes in serum creatinine rather than categorical KDIGO stages, enabling the detection of subclinical renal injury with greater sensitivity and capturing the full spectrum of postoperative renal stress.

Urinary CHIT1, KLK1 and CA3 demonstrated consistent performance across various age groups, identifying patients at risk of AKI by the end of surgery. Additionally, urinary KLK1 may aid preoperative risk stratification for AKI. These findings suggest potential improvements in diagnostic accuracy and quicker AKI detection, which could ultimately contribute to better long-term outcomes following congenital open-heart surgery.

As this was a hypothesis-generating study, these promising findings must be validated in future confirmatory studies. Such follow-up studies should include an a priori power analysis for the biomarkers of interest, involve more homogenous patient groups and incorporate established biomarkers, such as serum cystatin c and serum creatinine, as variables to check against AKI vs. no-AKI status.

## Figures and Tables

**Table 1:** Clinical characteristics and perioperative data.

	All Patients (n = 67)	AKI (n = 13)	Non-AKI (n = 54)	*p* Value
Age (years, IQR)	2 [0; 5]	0.5 [0.02; 20]	2 [0.5; 5.5]	0.31
Neonates (n, %)	6 (9.0)	4 (30.8)	2 (3.7)	**0.05**
Sex				0.58
Male (n, %)	42 (62.7)	8 (61.5)	34 (63.0)	
Female (n, %)	25 (37.3)	5 (38.5)	20 (37.0)	
Height (cm, IQR)	87.0 [64; 117]	71.0 [52.5;1 38.5]	87.5 [65; 120]	0.33
Weight (kg, IQR)	11.6 [5.7; 21]	7.81 [3.5; 47.5]	11.85 [6.0; 21.0]	0.31
Operation time (h, IQR)	5.4 [4.3; 7.1]	7.4 [5.8; 11.4]	4.9 [4.3; 6.2]	**0.001**
Aortic cross clamp time (min, IQR)	76.0 [29.0; 113.0]	91.0 [0.0; 175.5]	71.5 [30.5; 108.3]	0.28
CPB time (h, IQR)	2 [2.0; 3.2]	3.0 [2.0; 7.0]	2.0 [2.0; 3.0]	**0.04**
Minimal temperature (°C, IQR)	32.0 [28.2; 35.6]	30.0 [27.9; 33.9]	32.0 [28.9; 35.6]	0.067
Length of mechanical ventilation (h, IQR)	8.3 [6.3; 19.1]	51.9 [24.5; 251.6]	7.35 [6.1; 12.1]	**0.001**
Length of ICU stay (d, IQR)	1.9 [0.4; 4.4]	6.3 [4.6; 14.8]	1.3 [0.4; 2.4]	**0.001**
Length of hospital stay (d, IQR)	10.0 [8.0; 14.0	23.0 [15.0; 56.5]	9.0 [7.0;1 1.0]	**0.001**
VIS (24 h) mean (IQR)	2.7 [0.9; 6.8]	5.8 [4.4; 11.1]	2 [0.12; 5.94]	**0.04**
VIS (24 h) max (IQR)	6.4 [2.4; 13.6]	17.9 [7.8; 27.4]	4.8 [1.6; 9.7]	**0.001**
VIS (Total) mean (IQR)	2.5 [0.5; 4.9]	6.1 [3.7; 9.0]	1.3 [0.1; 4.1]	**0.001**
VIS (Total) max (IQR)	6.8 [2.4; 14.1]	17.8 [13.4; 27.5]	4.8 [1.6; 10.1]	**0.001**
Fluid balance (24 h, mL)	+630 [+282; +1256]	+680 [−328; +2189]	+583 [+290; +1172]	0.980
Fluid balance (48 h, mL)	−85 [−322; −207]	−81 [−454; +183]	−90 [−322; +230]	0.769
Fluid balance (72 h, mL)	−197 [−493; +58]	−156 [−433; +78]	−198 [−503; +60]	0.659
Administration of concentrated red blood cells (RBC) (mL, IQR)	120.0 [0; 250]	250.0 [100.0; 770.0]	100.0 [0; 200]	**0.003**
RAI-Score (IQR)	2.0 [1.0; 4.0]	20.0 [4.0; 20.0]	1.0 [1.0; 2.0]	**0.001**
RPP 15 min post CPB (mmHg, IQR)	48.0 [39.2; 61.8]	39.0 [35.0; 47.8]	52.6 [41.8; 63]	**0.05**
eGFR T0 (mL/min/m^2^, IQR)	76.4 [59.5; 89.5]	68.0 [35.3; 92.7]	76.0 [61.0; 89.6]	0.31
eGFR T1 (mL/min/m^2^, IQR)	74.3 [54.8; 90.0]	60.1 [35.3; 80.3]	76.7 [61; 90.4]	**0.04**
eGFR T2 (mL/min/m^2^, IQR)	77.4 [55.9; 92.4]	56.0 [27.0; 74.0]	80.5 [61.4; 95.9]	**0.003**
eGFR T3 (mL/min/m^2^, IQR)	71.9 [46.5; 88.8]	45.8 [24.0; 62.7]	76.4 [59.3; 95.3]	**0.001**

Continuous variables are given as median with [IQR], categorical variables are presented as counts and percentages (%). IQR = Interquartile range [Q1; Q3], AKI = acute kidney injury; CPB = cardiopulmonary bypass; VIS = vasoactive-inotropic score; RAI-Score = renal angina score; RBC = packed red blood cells; RPP = renal perfusion pressure; eGFR = estimated glomerular filtration rate; T0 = before surgery; T1 = direct after surgery; T2 = 6 h after surgery; T3 = 24 h after surgery.

**Table 2:** Laboratory parameters.

	All Patients (n = 67)	AKI (n = 13)	Non-AKI (n = 54)	*p* Value
Hb T0 (mg/dL, IQR)	13.6 [11.8; 15.2]	14.7 [13.1; 16.0]	13.3 [11.7; 15.0]	0.083
Hb T1 (mg/dL, IQR)	12.7 [11.1; 14.0]	13.1 [11.5; 16.3]	12.7 [10.9; 13.9]	0.236
Hb T2 (mg/dL, IQR)	12.4 [10.6; 14.5]	15.2 [11.0; 17.8]	12.1 [10.6; 13.5]	**0.023**
Hb T3 (mg/dL, IQR)	12.3 [10.5; 13.4]	14.2 [11.5; 16.4]	12.1 [10.2; 12.9]	**0.024**
Albumin T0 (g/dL, IQR)	3.8 [3.1; 4.0]	3.5 [2.9; 3.9]	4.0 [4.0; 4.0]	0.184
Albumin T1 (g/dL, IQR)	2.9 [2.5; 3.1]	2.0 [1.7; 2.1]	2.9 [2.6; 3.2]	0.002
Albumin T2 (g/dL, IQR)	2.0 [1.8; 2.8]	2.0 [1.9; 3.4]	2.1 [1.8; 2.4]	0.699
Albumin T3 (g/dL, IQR)	2.2 [2.1; 2.7]	2.2 [2.1; 3.0]	2.3 [2.0; 2.5]	0.722
sCr T0 (mg/dL, IQR)	0.53 [0.48; 0.61]	0.55 [0.43; 0.62]	0.53 [0.50; 0.56]	0.135
sCr T1 (mg/dL, IQR)	0.60 [0.53; 0.61]	0.60 [0.55; 0.62]	0.55 [0.50; 0.60]	**0.008**
sCr T2 (mg/dL, IQR)	0.65 [0.55; 0.78]	0.65 [0.60; 0.90]	0.55 [0.40; 0.70]	**<0.001**
sCr T3 (mg/dL, IQR)	0.80 [0.50; 0.98]	0.85 [0.56; 1.13]	0.65 [0.50; 0.8]	**<0.001**
sCr max (mg/dL, IQR)	0.87 [0.68; 1.22]	0.95 [0.62; 1.23]	0.87 [0.84; 0.90]	**<0.001**
Urea T0 (mg/dL, IQR)	21.3 [13.8; 33.7]	22.5 [9.5; 43.4]	21.3 [17.9; 24.8]	0.864
Urea T1 (mg/dL, IQR)	17.9 [10.8; 24.5]	15.5 [9.5; 34.0]	17.9 [17.0; 18.9]	0.480
Urea T2 (mg/dL, IQR)	22.7 [21.3; 36.7]	27.5 [21.3; 45.2]	22.5 [21.4; 23.5]	0.340
Urea T3 (mg/dL, IQR)	41.1 [30.2; 51.1]	43.9 [31.1; 62.0]	35.5 [31.2; 39.9]	**<0.001**
Potassium T0 (mmol/L, IQR)	3.8 [3.6; 4.1]	3.9 [3.4; 4.3]	4.1 [4.0; 4.1]	0.836
Potassium T1 (mmol/L, IQR)	3.9 [3.6; 4.2]	4.1 [3.0; 4.9]	4.3 [3.6; 5.0]	0.385
Potassium T2 (mmol/L, IQR)	4.0 [3.7; 4.2]	4.2 [3.5; 4.8]	4.4 [3.9; 4.8]	0.234
Potassium T3 (mmol/L, IQR)	3.8 [4.0; 4.2]	4.1 [3.9; 4.2]	4.5 [4.4; 4.5]	0.133
Sodium T0 (mmol/L, IQR)	138 [136; 139]	138 [135; 138]	140 [139; 141]	0.320
Sodium T1 (mmol/L, IQR)	144 [141; 146]	143 [139; 145]	147 [146; 148]	0.554
Sodium T2 (mmol/L, IQR)	147 [145; 149]	147 [145; 148]	148 [146; 151]	0.559
Sodium T3 (mmol/L, IQR)	147 [145; 149]	147 [146; 149]	147 [145; 149]	**0.002**
Glucose T0 (mg/dL, IQR)	94.5 [83; 103]	86.0 [64; 125]	91.5 [74; 109]	0.642
Glucose T1 (mg/dL, IQR)	129 [112; 149]	224 [156; 267]	127 [111; 142]	0.229
Glucose T2 (mg/dL, IQR)	125 [110; 142]	175 [119; 231]	127 [123; 131]	0.390
Glucose T3 (mg/dL, IQR)	127 [108; 151]	116 [112; 181]	150 [140; 160]	0.163

Continuous variables are given as median with [IQR]. IQR = Interquartile range [Q1; Q3], AKI = acute kidney injury; Hb = hemoglobin; sCr = serum creatinine; T0 = before surgery; T1 = direct after surgery; T2 = 6 h after surgery; T3 = 24 h after surgery. Creatinine clearance was not routinely measured in all patients and is therefore not reported comprehensively.

## Data Availability

The data supporting the findings of this study are available upon request from the corresponding author, R.S. However, the data are not publicly accessible as they contain information that could compromise the privacy of the research participants.

## Supplementary Materials

The following supporting information can be downloaded at: https://www.mdpi.com/article/10.3390/jcm14228253/s1, Table S1: Proteinselection; Figure S1: Changes in the proteome following surgery in patients with and without AKI

## Abbreviations

AKI	acute kidney injury
AUC	area under the curve
B2M	beta-2-microglobulin
CA3	carbonic anhydrase 3
CPB	cardiopulmonary bypass
CC	cellular component
CHIT1	chitotriosidase-1
CHMP5	charged multivesicular body protein 5
eGFR	estimated glomerular filtration rate
ICU	intensive care unit
IL-18	interleukin-18
IQR	interquartile range
KDIGO	kidney disease: improving global outcome.
KIM-1	kidney injury molecule-1
KLK1	kallikrein-1
L-FABP	liver type fatty acid-binding protein
LFQ	label free quantification
NGAL	neutrophil gelatinase-associated lipocalin
ROC	receiver operating characteristics
SCr	serum creatinin
T	Timepoint
TPM4	Tropomyosin alpha-4 chain
U	urinary
VIS	vasoactive-inotropic score
YIPF3	Protein YIPF3
