# Supplementary figures and images for: Early Detection of Acute Kidney Injury After Congenital Heart Surgery—Using Urine Proteomics to Identify New Biomarker Candidates: A Prospective Clinical Study
Source: J Clin Med. 2025 Nov 20;14(22):8253. doi: 10.3390/jcm14228253 (PMC12653219; doi:10.3390/jcm14228253)

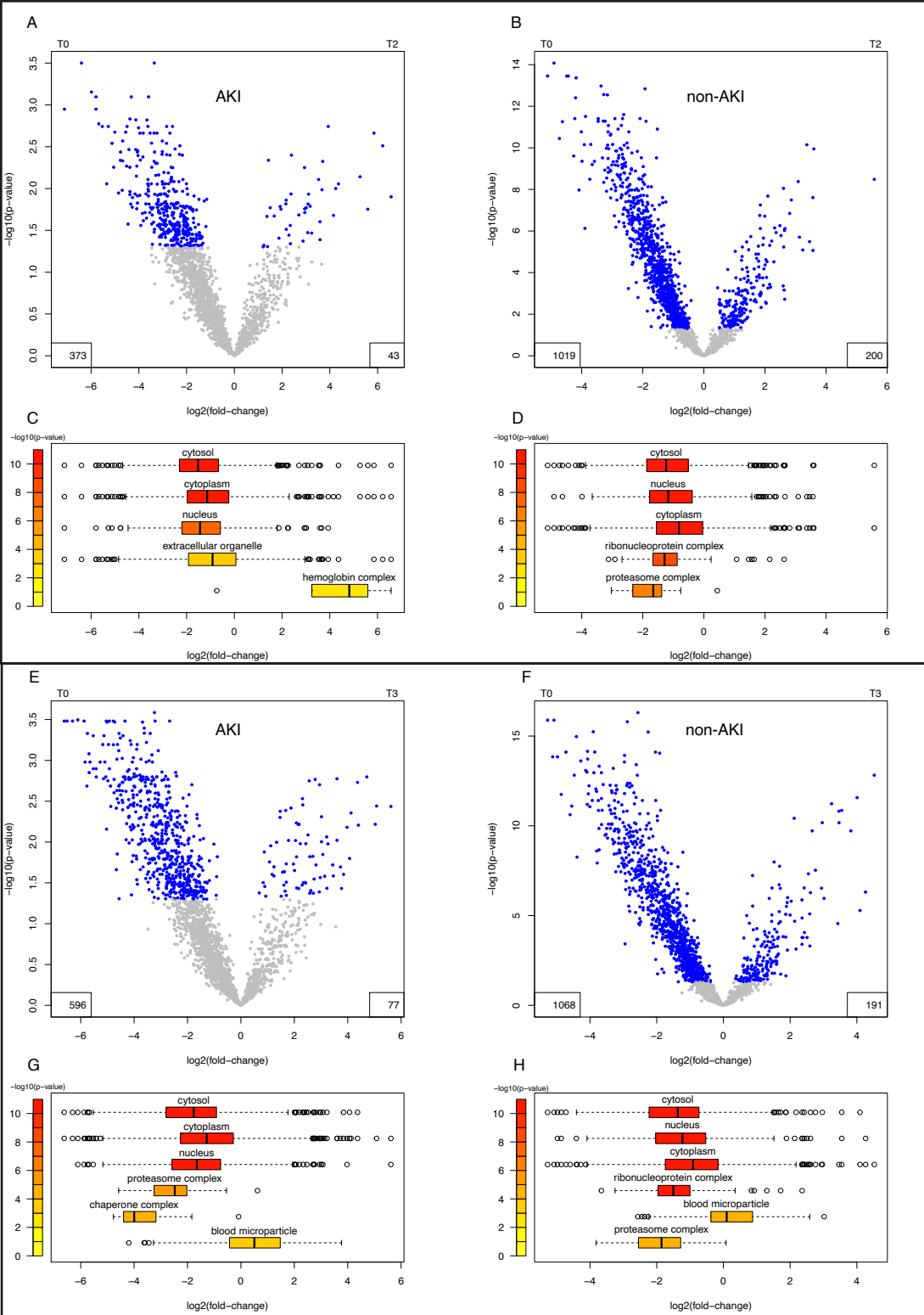

Supplement: Supplementary file 1 [file jcm-14-08253-s001.zip › Supplemental/Supplemental Figure 1.pdf]
